# Supplementary figures and images for: The GalNAc-T Activation Pathway (GALA) is not a general mechanism for regulating mucin-type O-glycosylation
Source: PLoS One. 2017 Jul 18;12(7):e0179241. doi: 10.1371/journal.pone.0179241 (PMC5515409; doi:10.1371/journal.pone.0179241)

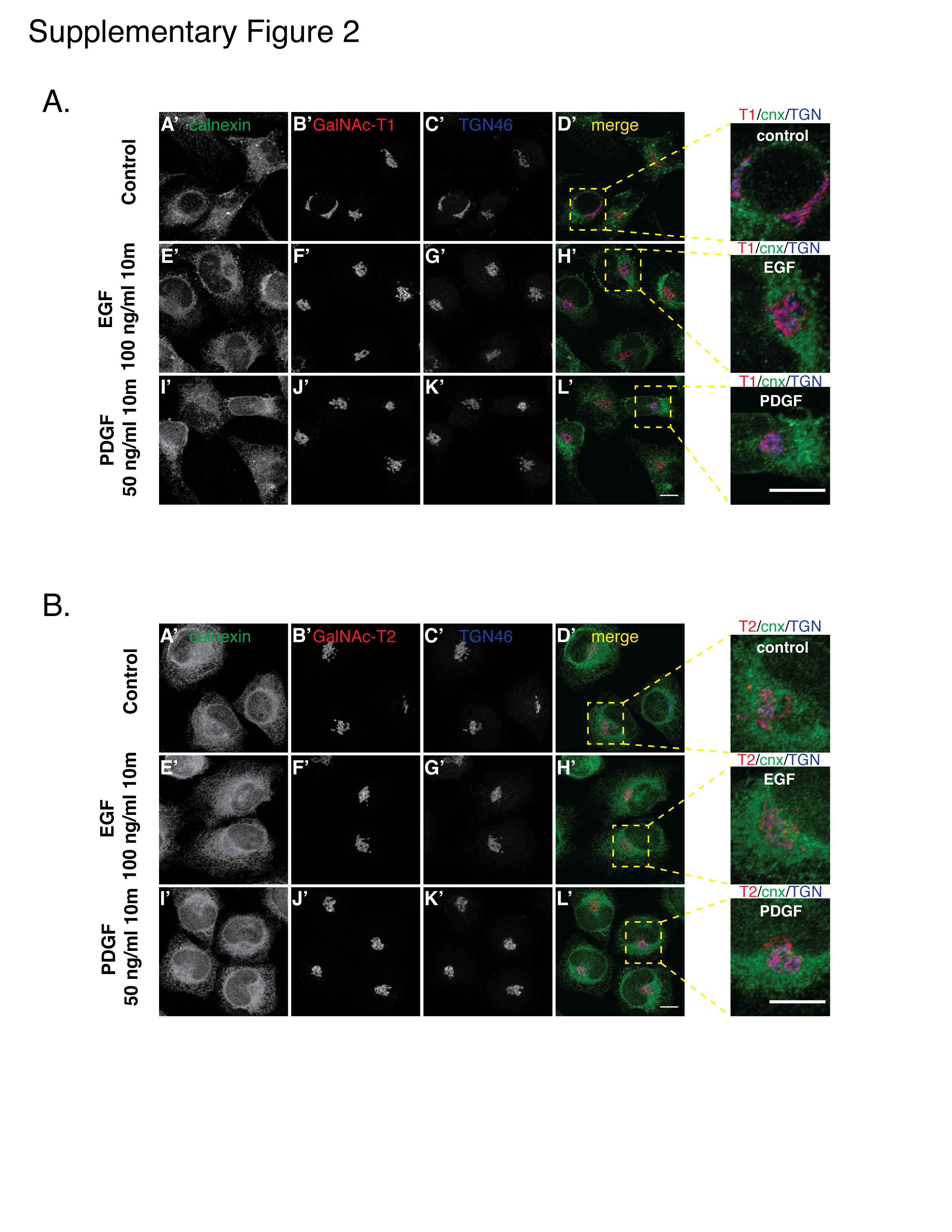

Supplement: S2 Fig — Serum starved HeLa cells were either left unstimulated, treated with 100 ng/ml of EGF for 10 min or 50 ng/ml of PDGF for 10 min. Cells were subsequently immunostained with antibodies to TGN46 (AC’, AG’, AK’, BC’, BG’ and BK’), the ER marker calnexin (AA’, AE’, AI’, BA’, BE’, and BI’) and either endogenous GalNAc-T1 (AB’, AF’, and AJ’) or GalNAc-T2 (BB’, BF’ and BJ’). Merged channels (AD’, AH’, AL’, BD’, BH’, and BL’) demonstrate that no change in GalNAc-T Golgi complex localization is observed. Individual maximum projections of 30 confocal sections shown in (A) are representative of 81, 85 and 82 cells for control, EGF and PDGF treatments, respectively, from two independent experiments. Representative images in (B) are from 103, 123 and 136 cells for control, EGF and PDGF treatments, respectively, from three independent experiments. Scale bars, 10 μm. (TIF) [file pone.0179241.s002.tif]

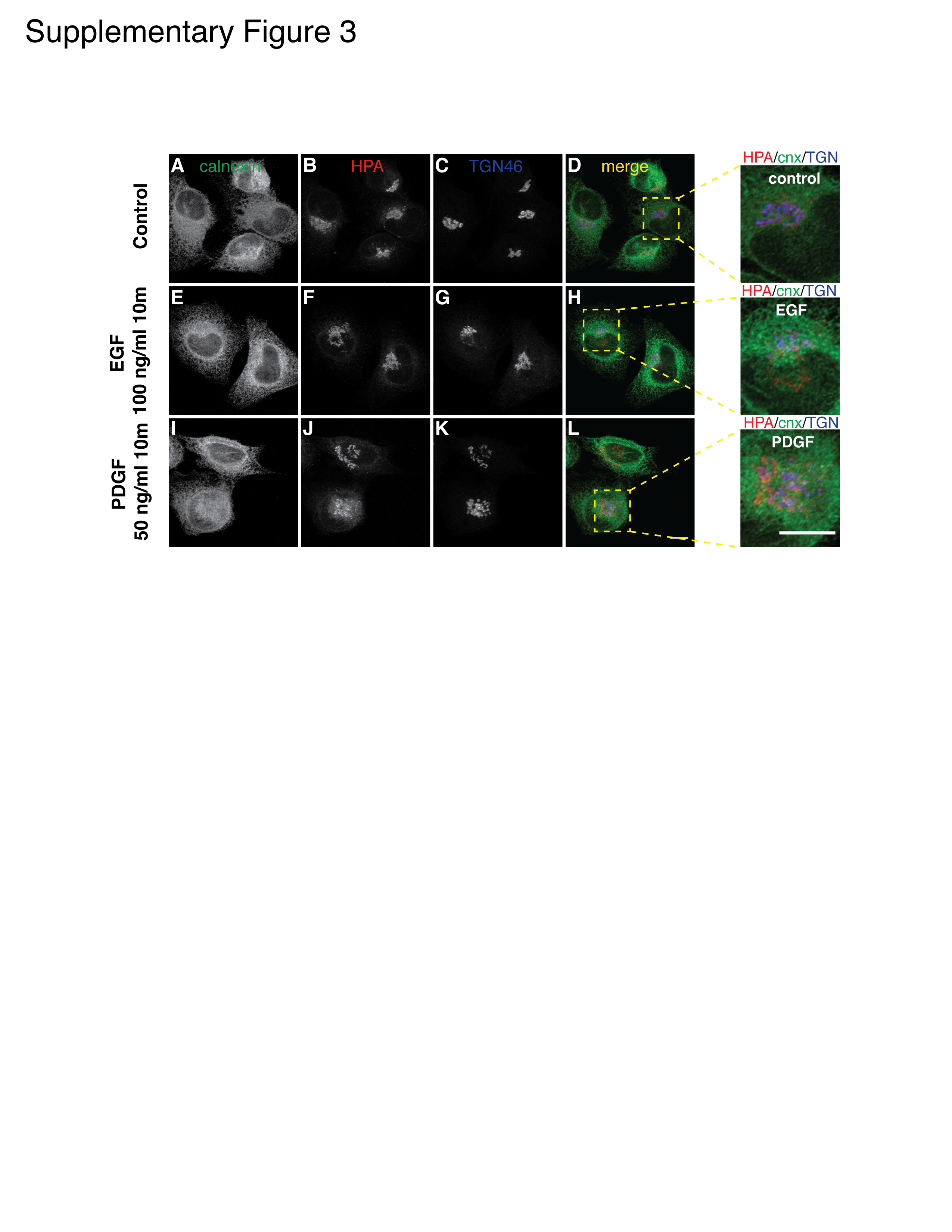

Supplement: S3 Fig — Serum starved HeLa cells were either left unstimulated, treated with 100 ng/ml of EGF for 10 min or 50 ng/ml of PDGF for 10 min. Cells were subsequently immunostained with antibodies to TGN46 (C, G and K), calnexin (A, E, and I) and the lectin HPA (B, F and J). Merged channels (D, H, and L) show that neither EGF nor PDGF treatment cause a change in the Golgi complex localization of Tn antigen. Individual maximum projections of 30 confocal sections are representative of 77, 91 and 93 cells for control, EGF and PDGF treatments, respectively, from three independent experiments. Scale bars, 10 μm. (TIF) [file pone.0179241.s003.tif]
